# Insights Into Spatial Orientation and Cognition in Tarantulas (Araneae: Theraphosidae) Under Natural Conditions, With Notes on Possible Ontogenetic Niche Shifts

**DOI:** 10.1002/ece3.73329

**Published:** 2026-03-30

**Authors:** Alireza Zamani, Rick C. West

**Affiliations:** ^1^ Zoological Museum, Department of Biodiversity Sciences University of Turku Turku Finland; ^2^ Sooke British Columbia Canada

**Keywords:** allothetic, idiothetic, learning, memory, troglobiont

## Abstract

Research on cognition in spiders, particularly in relation to navigation, has primarily focused on araneomorphs studied under controlled conditions. Mygalomorphs, such as tarantulas (Theraphosidae), have been largely neglected and almost nothing is known about their cognitive foraging behaviour in natural environments. Here, we present nine observations of arboreal and fossorial New World tarantulas, including a blind cave‐dwelling species, which together provide rare field‐based evidence that tarantulas may be capable of flexible, experience‐based navigation. All observed arboreal species, as well as two fossorial species, exhibited behaviour that may reflect spatial learning, by foraging in prey‐rich locations situated relatively far from their retreats. This behaviour differs from ontogenetic shifts in habitat use, which are noted here in several species for comparison; possible ontogenetic shifts in foraging behaviour in troglobitic tarantulas are also briefly discussed. The remaining observations involve tarantulas responding to disturbance with fast, direct returns to their burrows without disorientation. We discuss the likely allothetic and idiothetic cues underlying these behaviours, while also considering alternative or complementary explanations for retreat recognition and foraging movements based on chemical and chemo‐tactile cues. Finally, we briefly review existing experimental research on tarantula cognition, as well as studies on physiological and behavioural changes associated with stress or altered internal states that may interact with cognitive processes.

## Graphical abstract

Cognitive studies on spiders have largely ignored mygalomorphs, such as tarantulas, and almost completely lack field‐based observations. We present nine observations of arboreal and fossorial New World tarantulas, including a blind cave‐dwelling species, providing evidence for possible experience‐based navigation and spatial learning during foraging and retreat localisation.

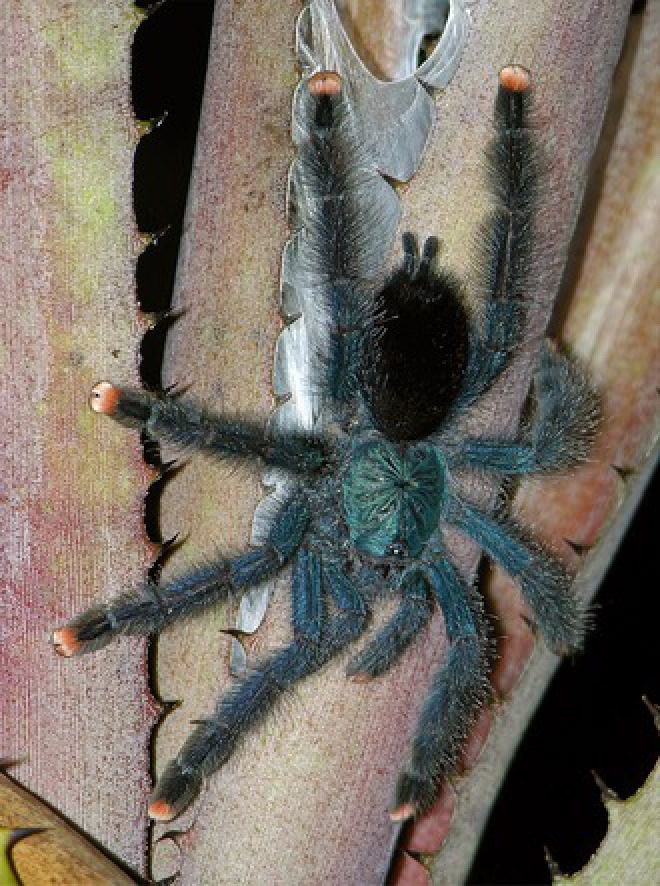

## 1 Introduction

Spatial orientation is the ability of an organism to perceive its position and body posture in three‐dimensional space and to use this information to guide movement, maintain balance and navigate within its environment. In spiders, spatial orientation relies on the integration of allothetic and idiothetic navigational cues. Idiothetic cues are self‐referential, that is, derived from the animal's own movements, including proprioceptive input from mechanosensory structures (Seyfarth et al. 1982), whereas allothetic cues are external, primarily visual, vibrational, or chemical.

These mechanisms function not only during short‐distance movements, such as quickly leaving a retreat to capture prey and returning along the same route, but also during nocturnal, long‐distance mate‐searching excursions that may extend tens of meters, followed by a direct homing trajectory back to the burrow (Nørgaard 2005). Of the three main homing strategies, that is, retracing the outbound path, using a ‘cognitive map’ based on geometric information from environmental landmarks, and path integration, the latter appears to be the primary strategy employed by spiders, involving the integration of distance and directional information acquired during the outbound journey (Gaffin and Curry 2020; Ortega‐Escobar 2020). It has been suggested that the complex path integration tasks reported in some species may involve the use of visual landmark cues and spatial memory, as observed in vertebrates; however, evidence supporting the presence of this mechanism in arthropods remains limited (Collett 2019; Foelix 2025).

Research on learning and memory in spiders, particularly in relation to navigation, is relatively recent and largely based on araneomorphs under controlled conditions (Barth 2002; Japyassu and Laland 2017; Punzo 2004). In tarantulas (Theraphosidae) and other mygalomorphs, studies have primarily focused on close‐range orientation, such as retreat recognition and behavioural adjustment near the burrow entrance, mediated by chemo‐tactile cues conveyed by silk. By contrast, the role of cognition in orientation, particularly in natural environments, remains almost entirely unexplored, even though a few experiments suggest that tarantulas are capable of learning, retaining, and utilising information acquired through visual, chemical, and mechanosensory experiences under controlled conditions (Punzo 1988; Punzo 2002).

**FIGURE 1:**
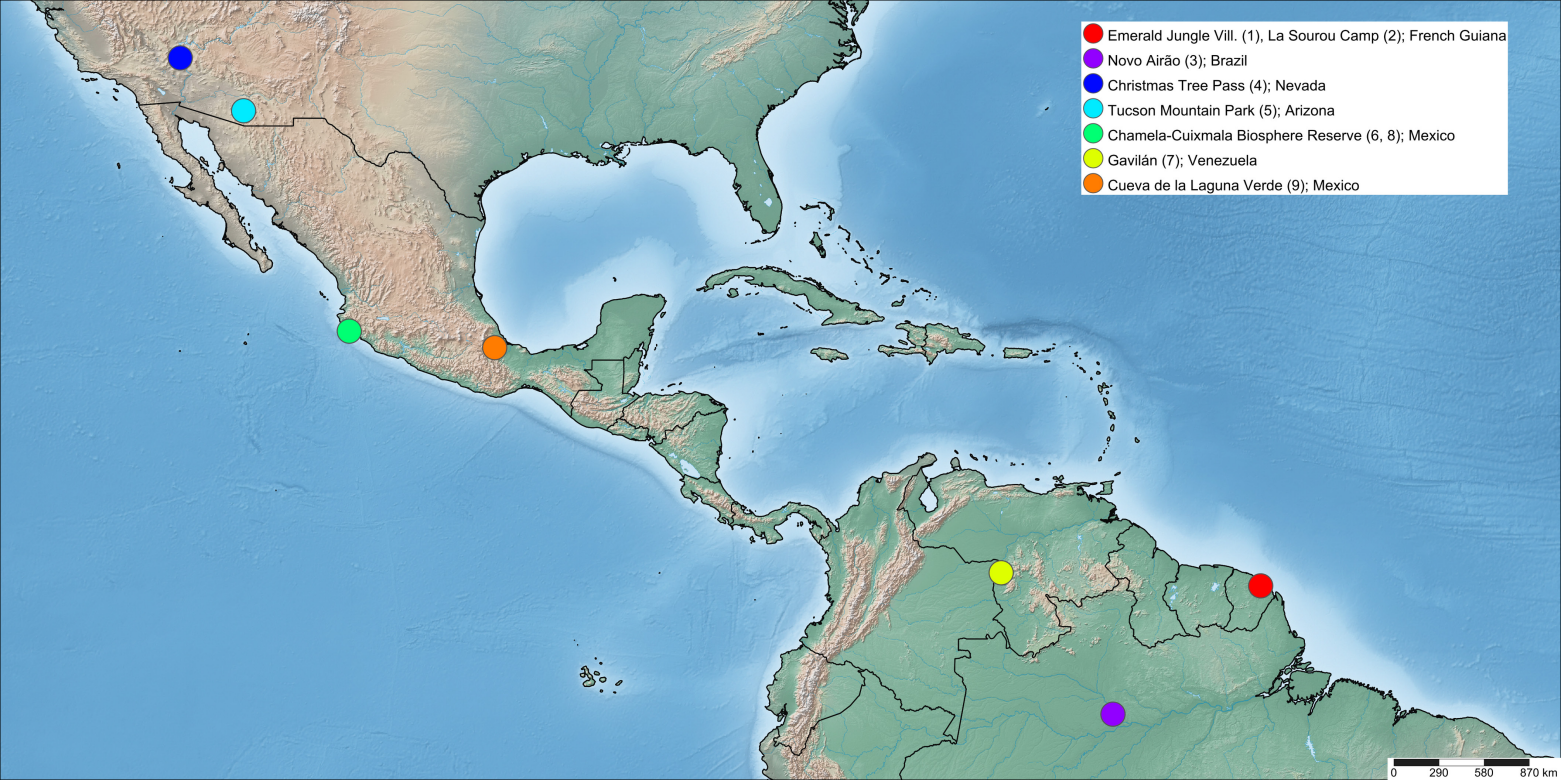
Partial map of the Americas showing localities of the nine observations reported here (coloured circles); map generated using SimpleMappr (Shorthouse 2010).

During independent field surveys across North and South America (Figure 1), RCW and other theraphosid researchers observed behaviours in juveniles (J), subadults (SA), and adults (A) of multiple species that may indicate cognitive involvement in routine foraging away from their retreats. Here, we describe these observations and discuss the possible roles of learning and memory in shaping such behaviours. Several cases of ontogenetic shifts, either in microhabitat use or foraging behaviour, are also briefly discussed.

## 2 Observations

### 2.1 Arboreal Tarantulas (Aviculariinae)

**FIGURE 2:**
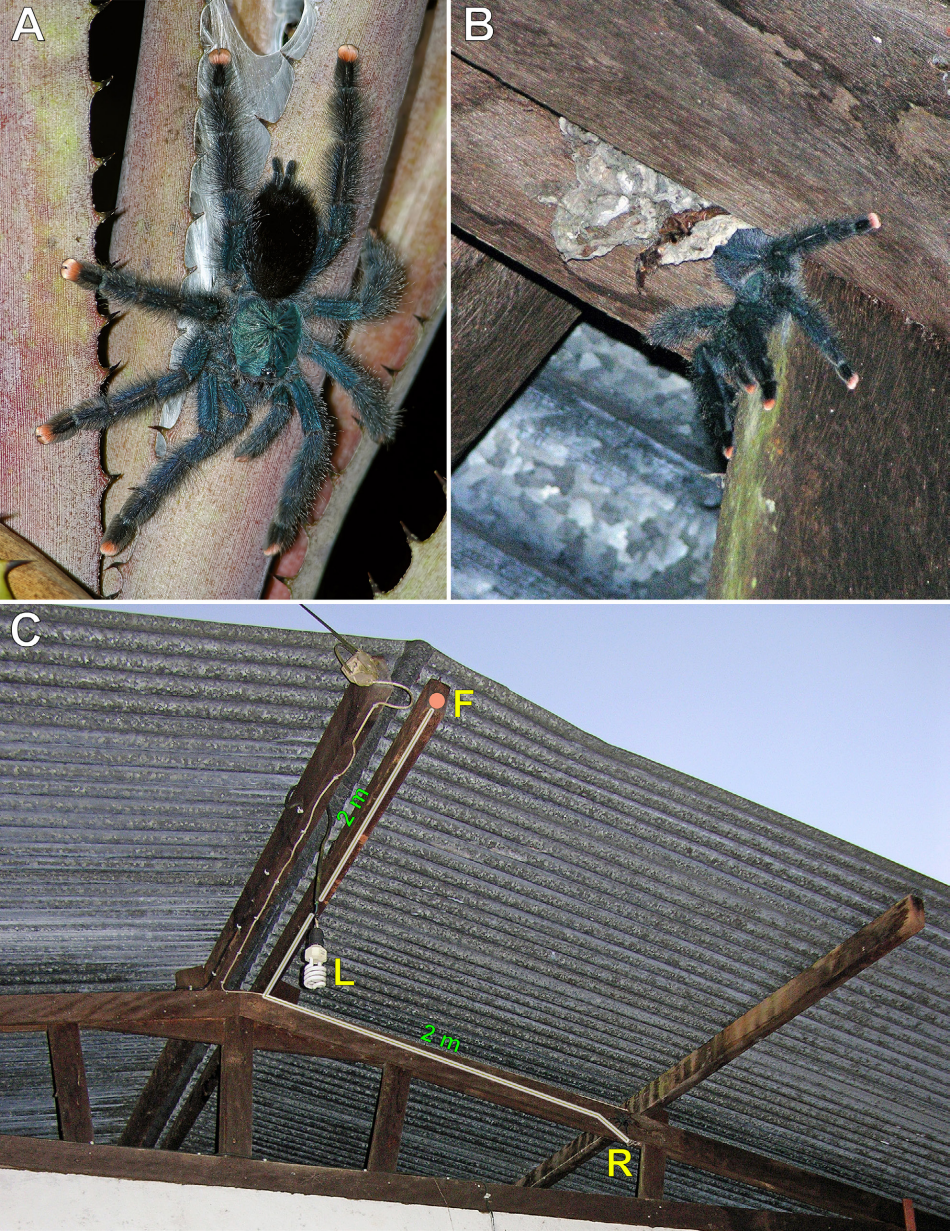
*Avicularia avicularia* , adult female; Emerald Jungle Village, Montsinéry, French Guiana. (A) Individual in a downward‐facing posture. (B) Individual emerging from its silken retreat constructed between wooden beams on the underside of a lodge roof. (C) Nocturnal movement route along the beams between the retreat (R) and the foraging site (F) adjacent to a light bulb (L). Photos: Rick C. West.

**Observation #1** (Figure 2A–C): *Avicularia avicularia* (Linnaeus, 1758) [A; ♀]; Emerald Jungle Village [4°47′05.0″N 52°25′21.0″W], Montsinéry, French Guiana; 09.2004; R.C. West.

For two consecutive weeks, every night, this individual (Figure 2A) was observed leaving its silken retreat (R), constructed between wooden beams on the underside of the lodge roof, shortly after sunset (Figure 2B,C). The tarantula travelled approximately 2 m along the underside of one beam, then made a right‐angle turn to its left and proceeded another 2 m to a position at the end of the beam, where it was observed foraging (F) and consuming flying insects (mainly large moths) attracted to the light (L) suspended beneath the roof beam (Figure 2C).

**FIGURE 3:**
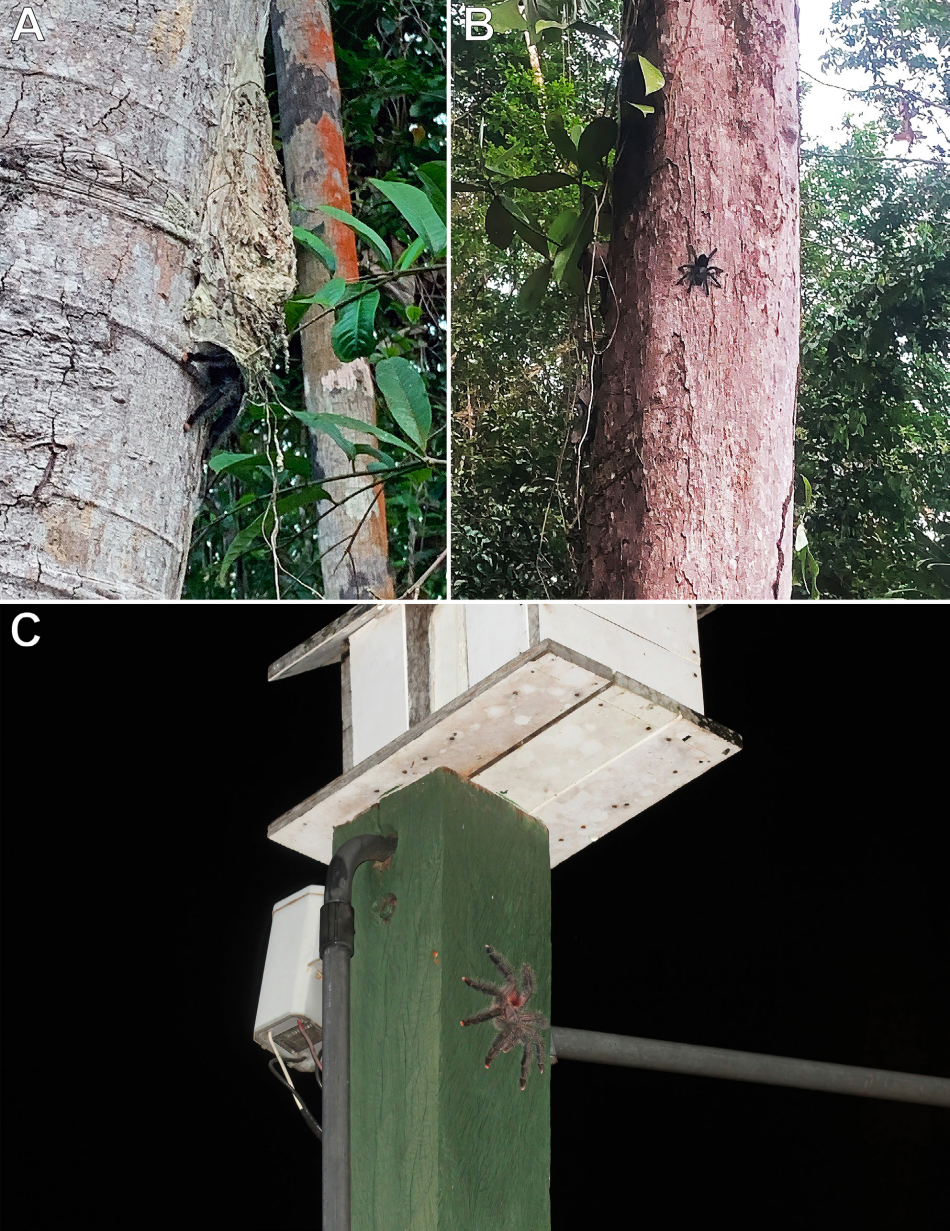
*Avicularia* spp., adult or subadult females. (A,B) *A. avicularia* , La Sourou Camp, Roura, French Guiana: (A) individual positioned at the entrance of its silken retreat on the trunk of a tall tree; (B) individual in a downward‐facing posture. (C) *A. variegata* , individual in a downward‐facing posture; Novo Airão, Amazonas, Brazil. Photos: Caroline Leblond and Dominique Visser (A,B), and Rogério Bertani (C).

**Observation #2** (Figure 3A,B): *A. avicularia* [SA/A; ♀]; La Sourou Camp [4°39′52.7″N 52°21′20.5″W], Roura, French Guiana; 04.2025; C. Leblond, D. Visser.

On multiple occasions, this individual was observed leaving its silken retreat constructed on the east side of a tall tree (Figure 3A), walking approximately 2 m down from the retreat before positioning itself in a downward‐facing posture to wait for prey (Figure 3B).

**Observation #3** (Figure 3C): *Avicularia variegata* F.O. Pickard‐Cambridge, 1896 [SA/A; ♀]; Novo Airão [2°37′23.9″S 60°56′35.3″W], Amazonas, Brazil; 11.2017; R. Bertani.

This individual was observed emerging at night from its silken retreat, constructed inside an unused birdhouse mounted atop a tall pole. The tarantula navigated around the far side of the birdhouse and down the green pole before positioning itself approximately 1 m away in a downward‐facing stance to wait for and capture prey in an area illuminated by a light mounted at the end of the metal conduit pipe on the right (Figure 3C).

### 2.2 Fossorial Tarantulas (Theraphosinae)

**FIGURE 4:**
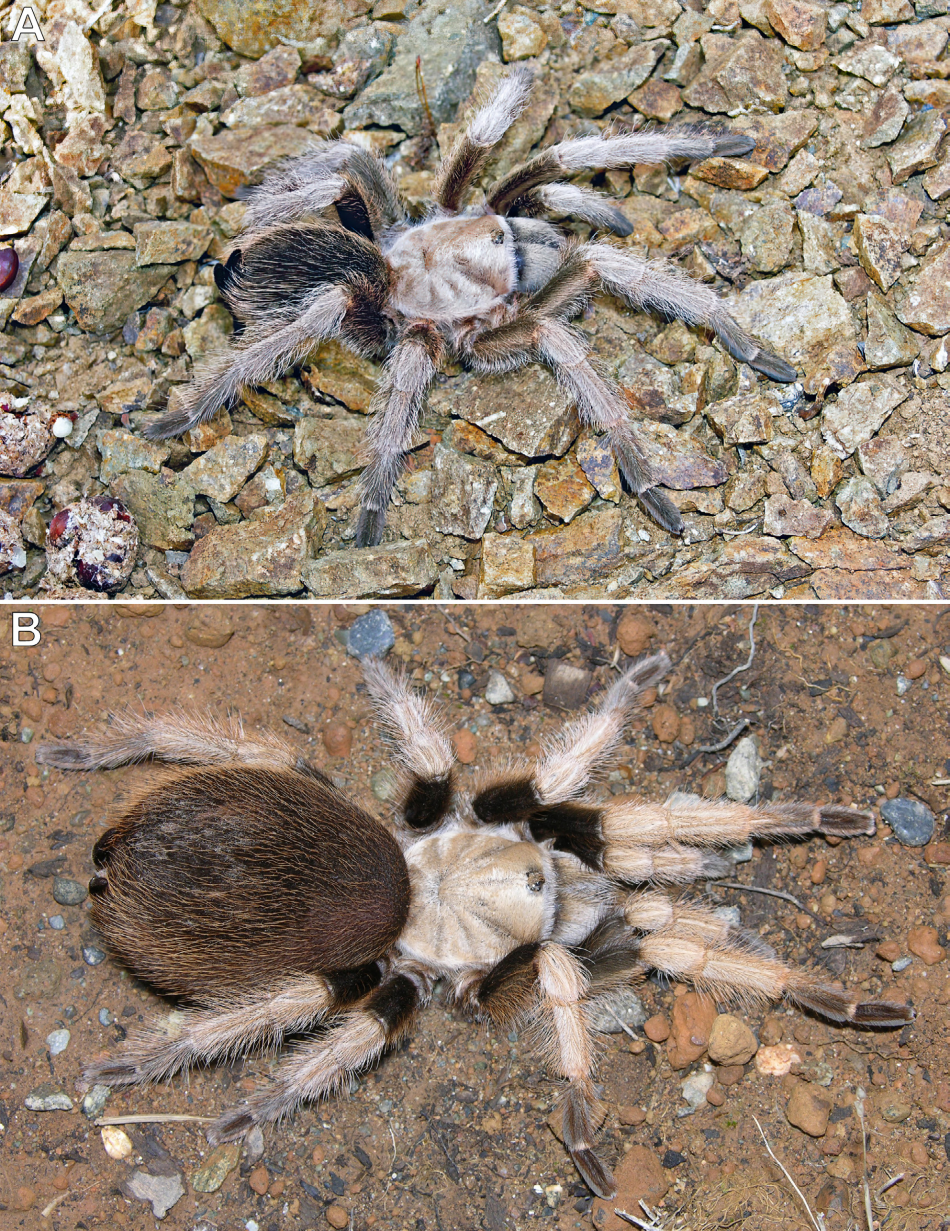
*Aphonopelma* spp., adult females. (A) *A. iodius* ; Christmas Tree Pass, Clark County, Nevada, USA. (B) *A. chalcodes* ; Tucson Mountain Park, Pima County, Arizona, USA. Photos: Rick C. West.

**FIGURE 5:**
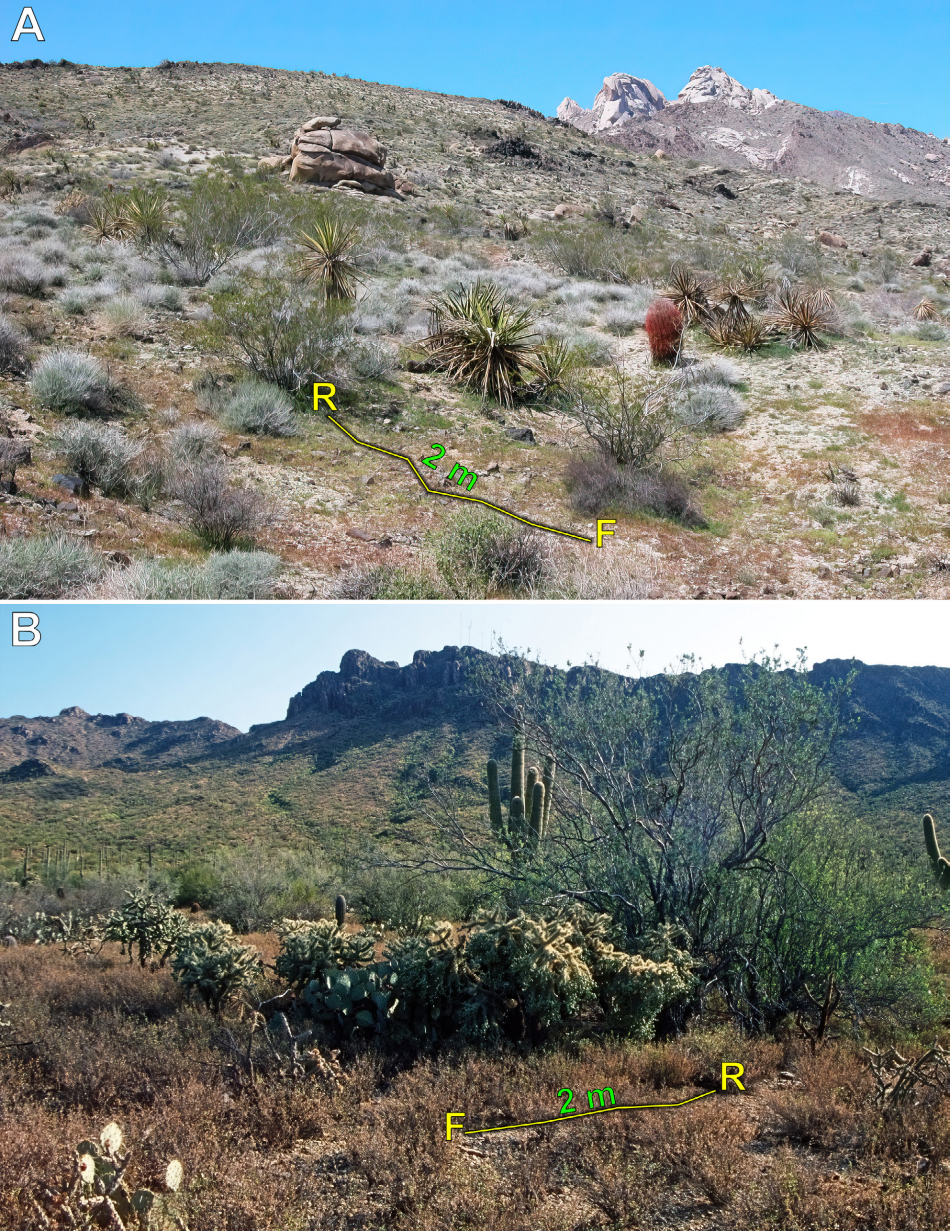
Daytime view of the nocturnal movement route from the presumed foraging site (F) to the burrow (R) of *Aphonopelma* spp. (A) *A. iodius* ; Christmas Tree Pass, Clark County, Nevada, USA. (B) *A. chalcodes* ; Tucson Mountain Park, Pima County, Arizona, USA. Photos: Rick C. West.

**FIGURE 6:**
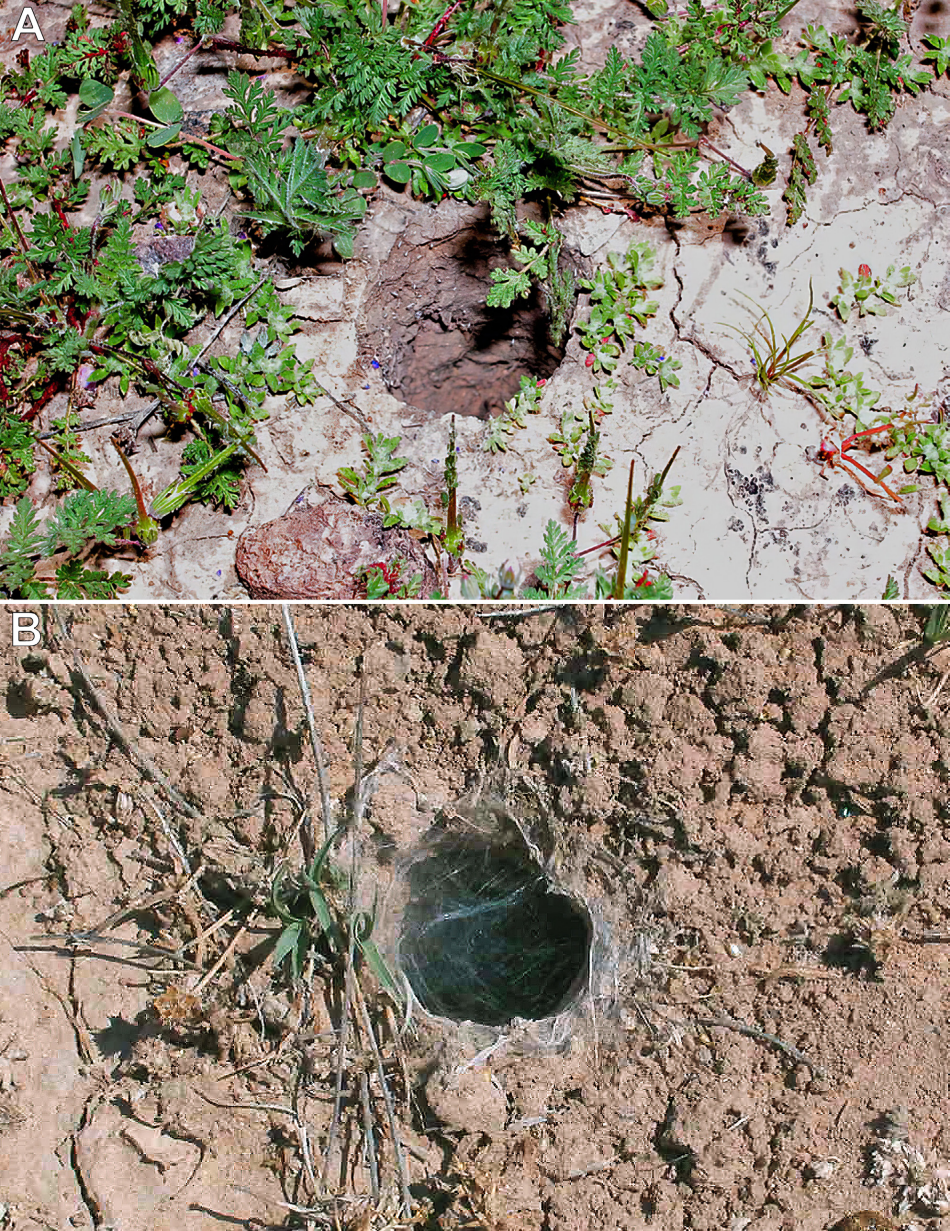
Fossorial burrows of *Aphonopelma* spp. (A) *A. iodius* , burrow at the base of a creosote bush amid sparse vegetation; Christmas Tree Pass, Clark County, Nevada, USA. (B) *A. chalcodes* , burrow near low grasses; Tucson Mountain Park, Pima County, Arizona, USA. Photos: Rick C. West.

**Observation #4** (Figures 4A, 5A, 6A): *Aphonopelma iodius* (Chamberlin & Ivie, 1939) [A; ♀]; Christmas Tree Pass [35°15′35.4″N 114°44′43.8″W], Clark County, Nevada, USA; 04.2003; R.C. West.

The spider (Figure 4A) was observed on the ground at night, presumably foraging for prey (F). When approached, either advancing footstep vibrations or the increased intensity of the flashlight beam triggered a quick retreat of approximately 2 m back to its burrow (R), along a relatively straight path (Figures 5A, 6A). The approximate nocturnal route taken back to the burrow was photographed the following day.

**Observation #5** (Figures 4B, 5B, 6B): *Aphonopelma chalcodes* Chamberlin, 1940 [A; ♀]; Tucson Mountain Park [32°12′48.1″N 111°05′18.1″W], Pima County, Arizona, USA; 09.2012; R.C. West.

While searching for tarantulas and scorpions along a trail at night using a flashlight, this individual (Figure 4B) was observed standing motionless on the ground, presumably foraging (F). When approached, advancing footstep vibrations or increased flashlight intensity likely triggered a quick retreat back to its burrow (R) along a relatively straight path of approximately 1.5 m (Figures 5B, 6B). The approximate nocturnal route taken back to the burrow was photographed the following day.

**FIGURE 7:**
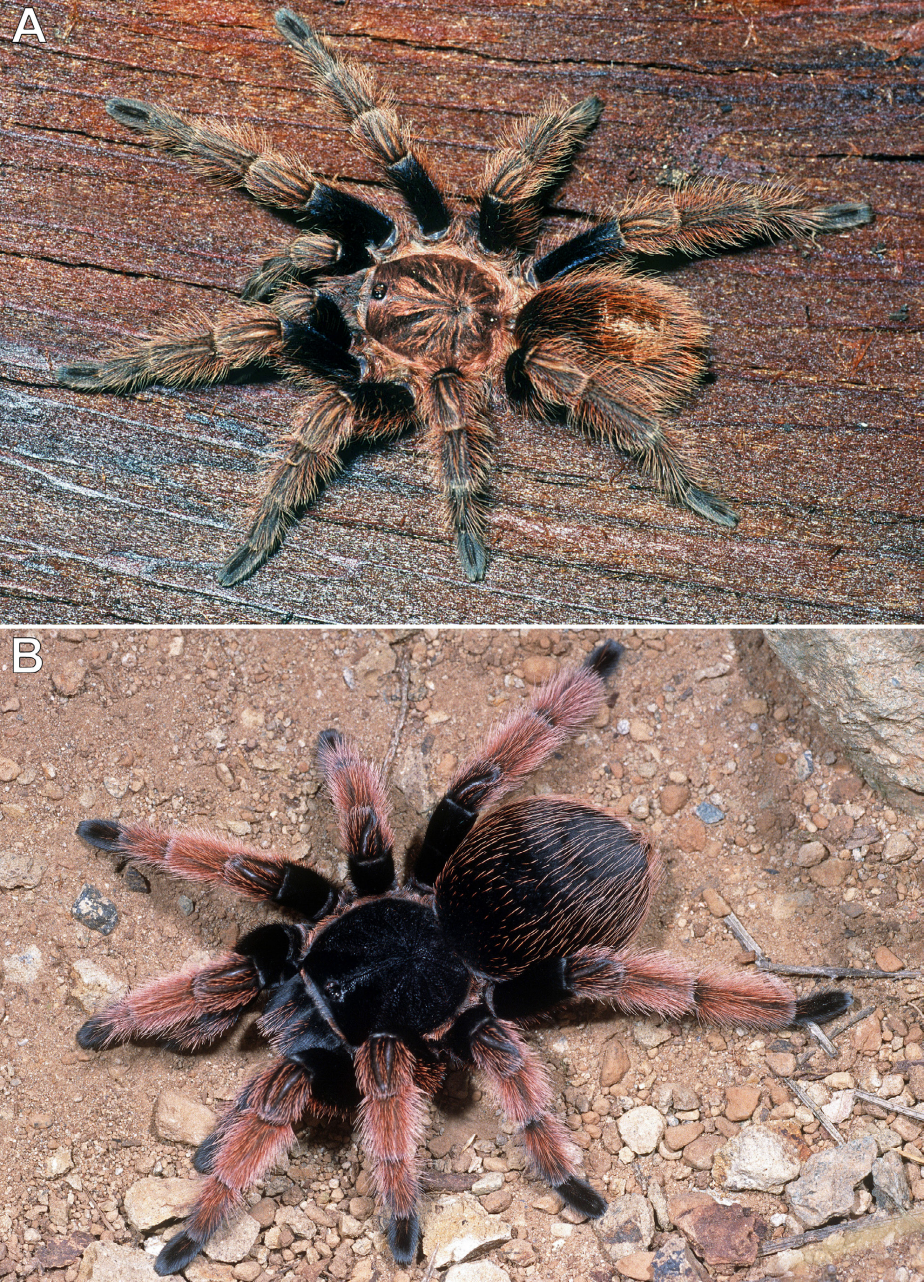
*Bonnetina* cf. *cyaneifemur* (A) and *Brachypelma klaasi* (B), adult or subadult females; Chamela‐Cuixmala Biosphere Reserve, Chamela, Jalisco, Mexico. Photos: Rick C. West.

**Observations #6** (Figure 7A,B): *Bonnetina* cf. *cyaneifemur* Vol, 2000 and *Brachypelma klaasi* (Schmidt & Krause, 1994) [SA, A; ♀♀]; Chamela‐Cuixmala Biosphere Reserve [19°29′17.2″N 104°59′44.0″W], Chamela, Jalisco, Mexico; 7–9.2022; D.C. Ramírez.

During nocturnal field studies conducted in the dry season, a subadult or adult *B*. cf. *cyaneifemur* (Figure 7A), and on multiple occasions subadult or adult individuals of *B. klaasi* (Figure 7B), were observed ranging approximately 0.5–1 m away from their ground burrows, presumably hunting for prey. When disturbed, all encountered tarantulas retreated quickly and directly back to their burrows without difficulty or hesitation.

**FIGURE 8:**
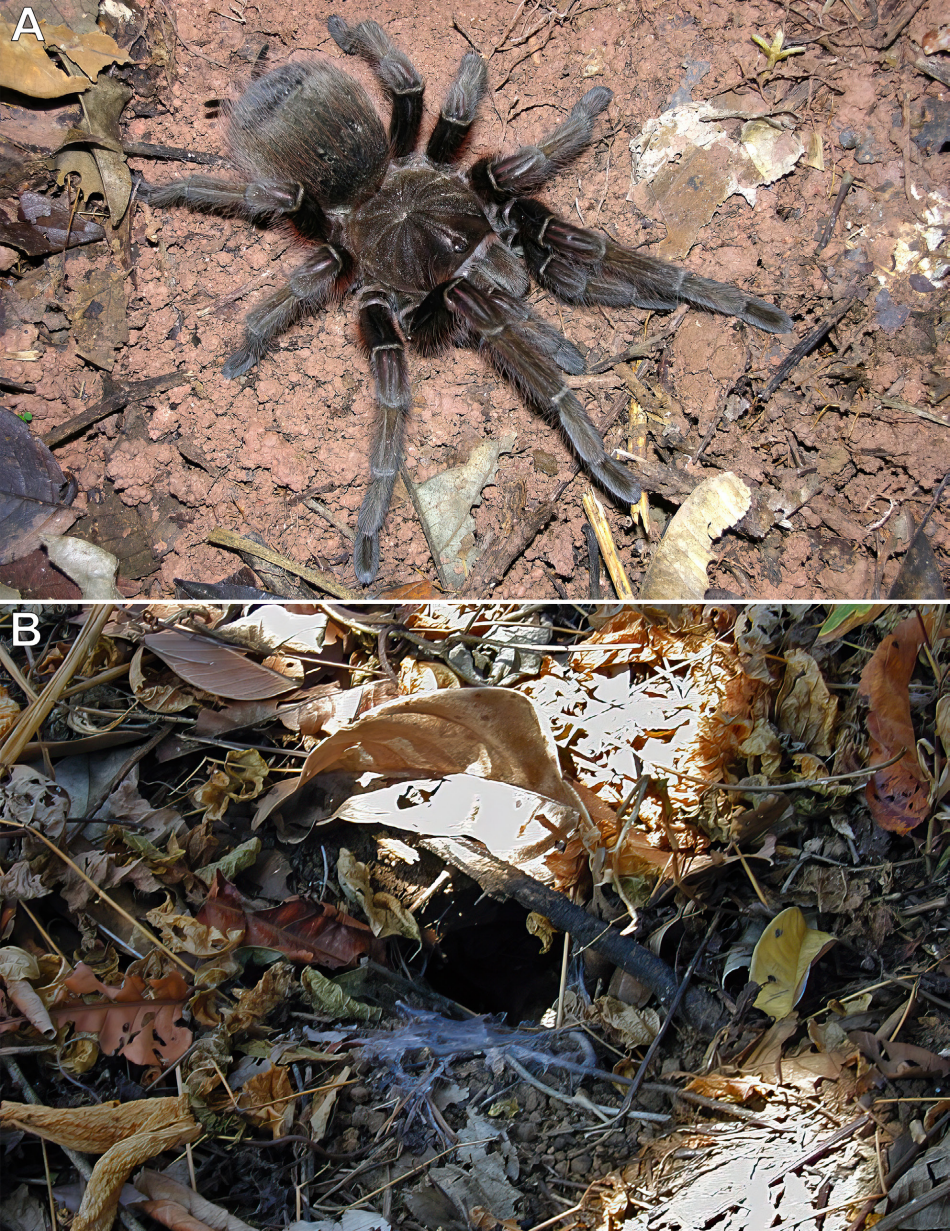
*Theraphosa apophysis* , adult female and burrow; Gavilán, Amazonas, Venezuela. (A) Adult female. (B) Fossorial burrow among leaf litter. Photos: Rick C. West.

**FIGURE 9:**
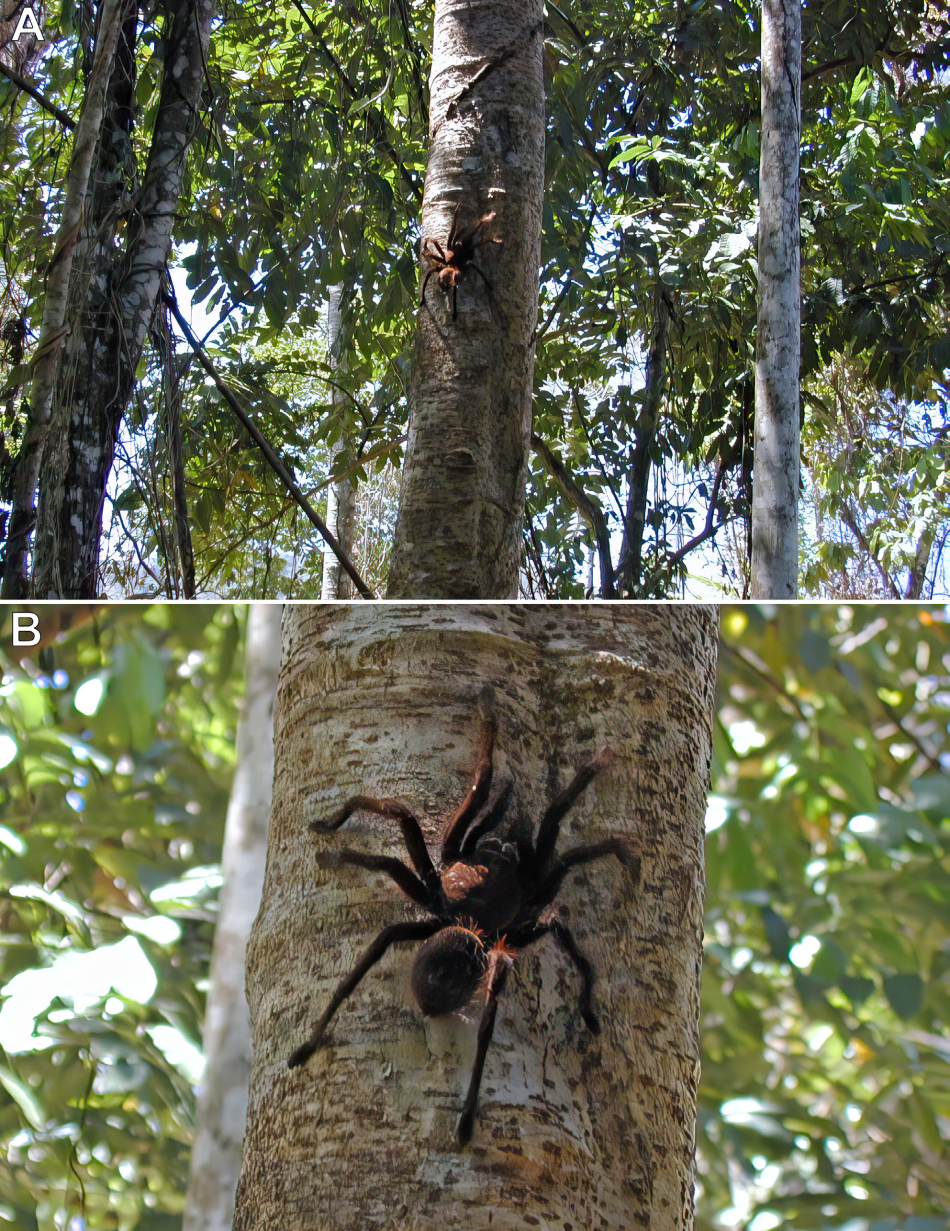
*Theraphosa apophysis* , adult female; Gavilán, Amazonas, Venezuela. (A) Individual climbing approximately 12 m up a tall tree toward the canopy foliage. (B) Closer view of the same individual. Photos: Rick C. West.

**Observation #7** (Figures 8A,B, 9A,B): *Theraphosa apophysis* (Tinter, 1994) [A; ♀]; Gavilán [5°32′46.9″N 67°23′22.2″W], Amazonas, Venezuela; 02.2002; R.C. West.

The spider (Figure 8A) was observed leaving its ground burrow (Figure 8B) on an overcast day during the dry season and climbing an adjacent tall tree toward the foliage canopy, reaching an estimated height of approximately 12 m above the ground (Figure 9A,B). Further observation was not possible due to time constraints. The tarantula may have been foraging in the tree canopy during the dry season.

**Observation #8**: *B. klaasi* [A; ♀]; Chamela‐Cuixmala Biosphere Reserve [19°29′17.2″N 104°59′44.0″W], Chamela, Jalisco, Mexico; 06.1997; M. Yáñez.

Similar to observation #7, during a day in the dry season, this individual was observed climbing down the side of a large acacia tree and returning to a nearby ground burrow. It was inferred that the tarantula, normally fossorial and nocturnal like *T. apophysis* , had been searching for prey during daylight hours in the dry season.

**FIGURE 10:**
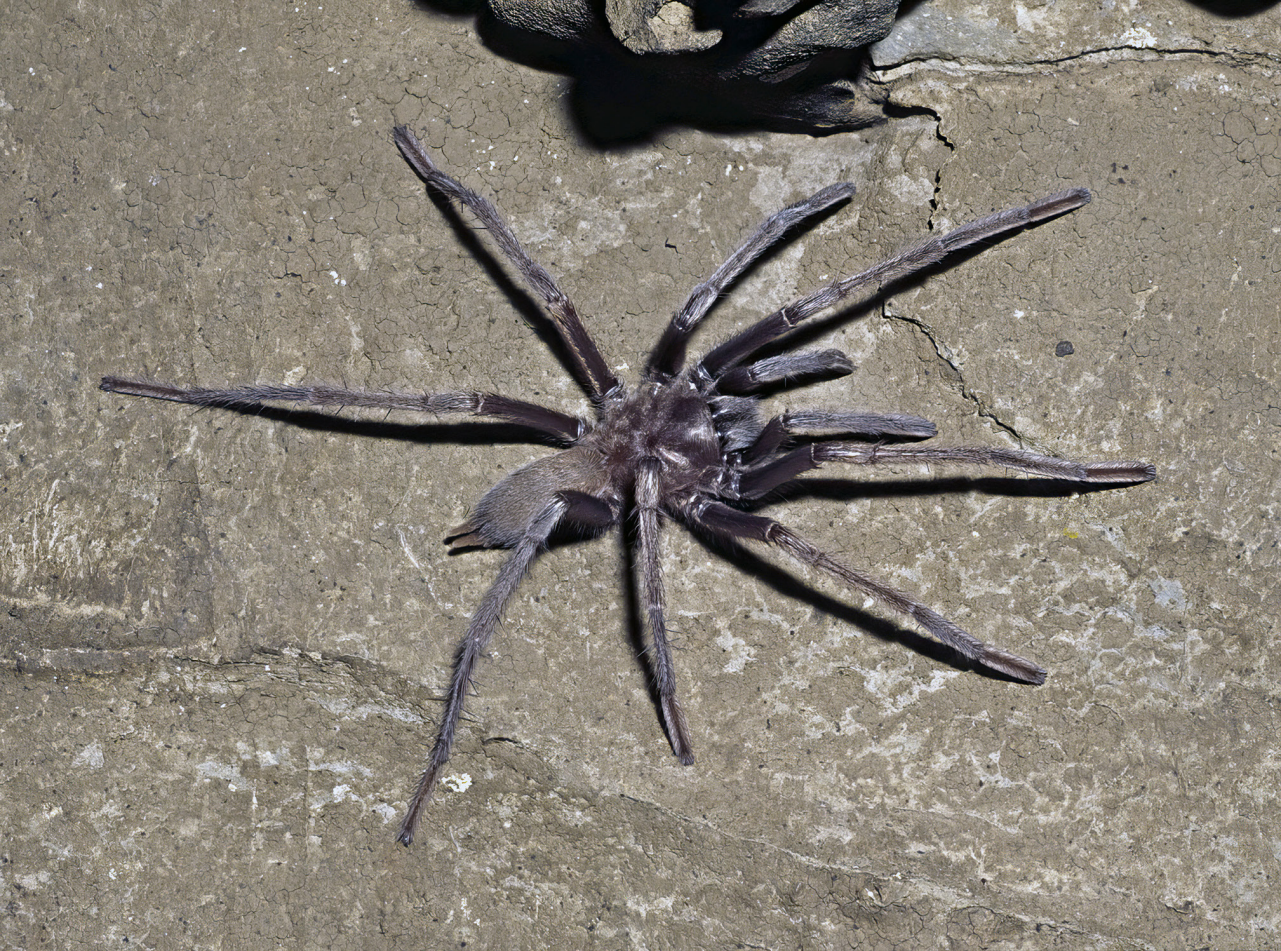
*Hemirrhagus sprousei* , adult female; Cueva de la Laguna Verde, Acatlán, Oaxaca, Mexico. Photo: Rick C. West.

**FIGURE 11:**
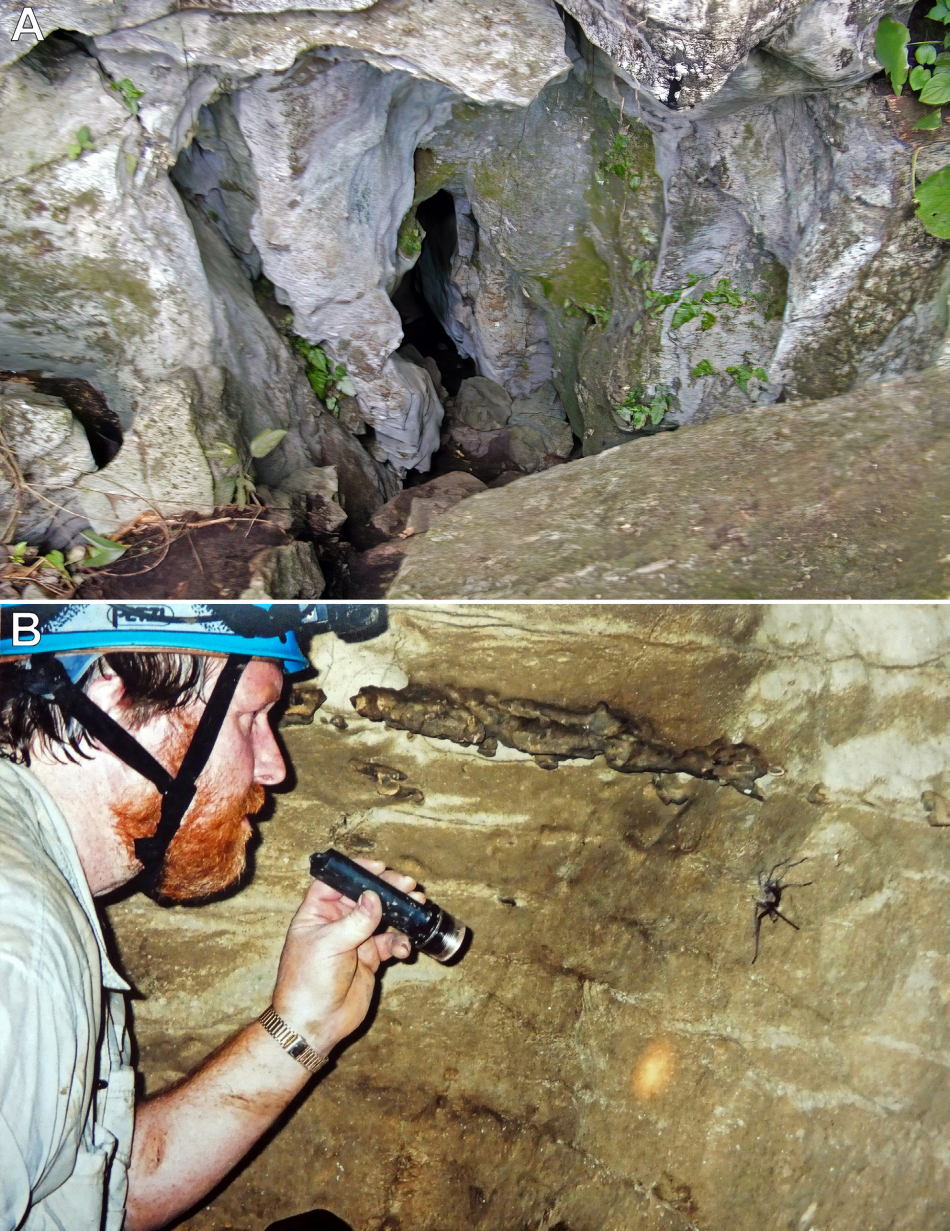
Cueva de la Laguna Verde, Acatlán, Oaxaca, Mexico. (A) One of the cave entrances. (B) Rick C. West observing a female *Hemirrhagus sprousei* foraging on a cave wall. Photos: Rick C. West (A) and Peter Sprouse (B).

**FIGURE 12:**
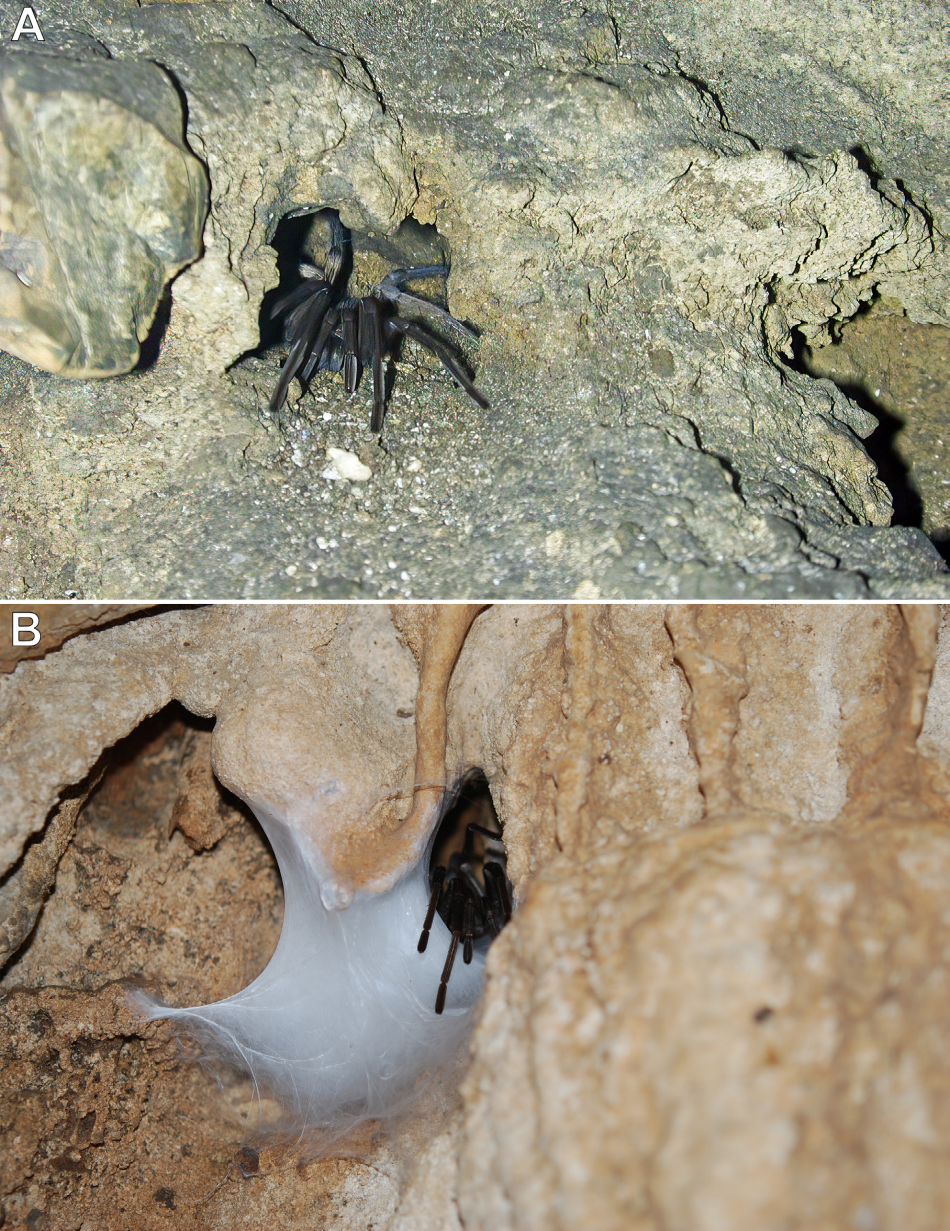
*Hemirrhagus* spp., adult females. (A) *H. sprousei* , individual inside a natural cavity retreat near the cave floor; Cueva de la Laguna Verde, Acatlán, Oaxaca, Mexico. (B) *H. papalotl* Pérez‐Miles & Locht, 2003, individual with a fixed, hammock‐like egg sac; Gruta de Zacatecolotla, Taxco de Alarcón, Guerrero, Mexico. Photos: Rick C. West (A) and Jorge Mendoza (B).

**Observation #9** (Figures 10, 11A,B, 12A): *Hemirrhagus sprousei* Mendoza & Francke, 2018 [J, SA, A; ♀♀]; Cueva de la Laguna Verde [18°32’N 96°36’W], Acatlán, Oaxaca, Mexico; 02.2002; R.C. West.

Cueva de la Laguna Verde (Green Lagoon Cave) is a 3350‐m‐long cave formed in water‐soluble limestone (karst) by underground streams (Figure 11A). The cave was visited specifically during the dry season to film the troglobitic tarantula *H. sprousei* (Figure 10), when stream water levels were low and the cave was more easily navigable. The cave also hosts several other endemic or narrow‐ranged species, including the blind crayfish *Procambarus oaxacae reddelli* Hobbs, 1973 (Cambaridae); a translucent prawn of the genus *Macrobrachium* Spence Bate, 1868 (Palaemonidae); millipedes of the genera *Prostemmiulus* Silvestri, 1916 (Stemmiulidae) and *Rhysodesmus* Cook, 1895 (Pyrgodesmidae); a scorpion of the genus *Vaejovis* Koch, 1836 (Vaejovidae); a blind catfish of the genus *Rhamdia* Bleeker, 1858 (Heptapteridae); and the robber frog *Craugastor decoratus* (Taylor, 1942) (Craugastoridae) (Coons 1976; Reddell 1981; Elliott 2020; Francke, pers. comm.; Sprouse, pers. comm.; West, pers. obs.).

Tarantulas were encountered approximately 300 m into the cave system, well beyond the light‐transition zone, where polarised light might otherwise have an additional effect on navigational behaviour in the cave's lightless interior. Unlike non‐troglobitic tarantulas, *H. sprousei* lacks an eye tubercle and possesses reduced eye cells that may be non‐functional, along with long, slender legs that are less hirsute than those of non‐troglobitic congeners.

Individuals were found on cave walls (Figure 11B), either just above the stream on the cave floor (Mendoza & Sprouse, pers. comm.; West, pers. obs.) or at the entrances of natural cavity retreats lacking a silk lining and situated above the flood zone (Figure 12A). When approached, they were highly sensitive to even slight ground vibrations (e.g., walking) or air currents (e.g., breathing) produced by nearby observers. Juveniles were observed at varying distances from burrows constructed in mud embankments above the stream's high‐water level (Sprouse, pers. comm.). When disturbed, these individuals quickly and directly returned to their retreats without hesitation or disorientation.

## 3 Discussion

**FIGURE 13:**
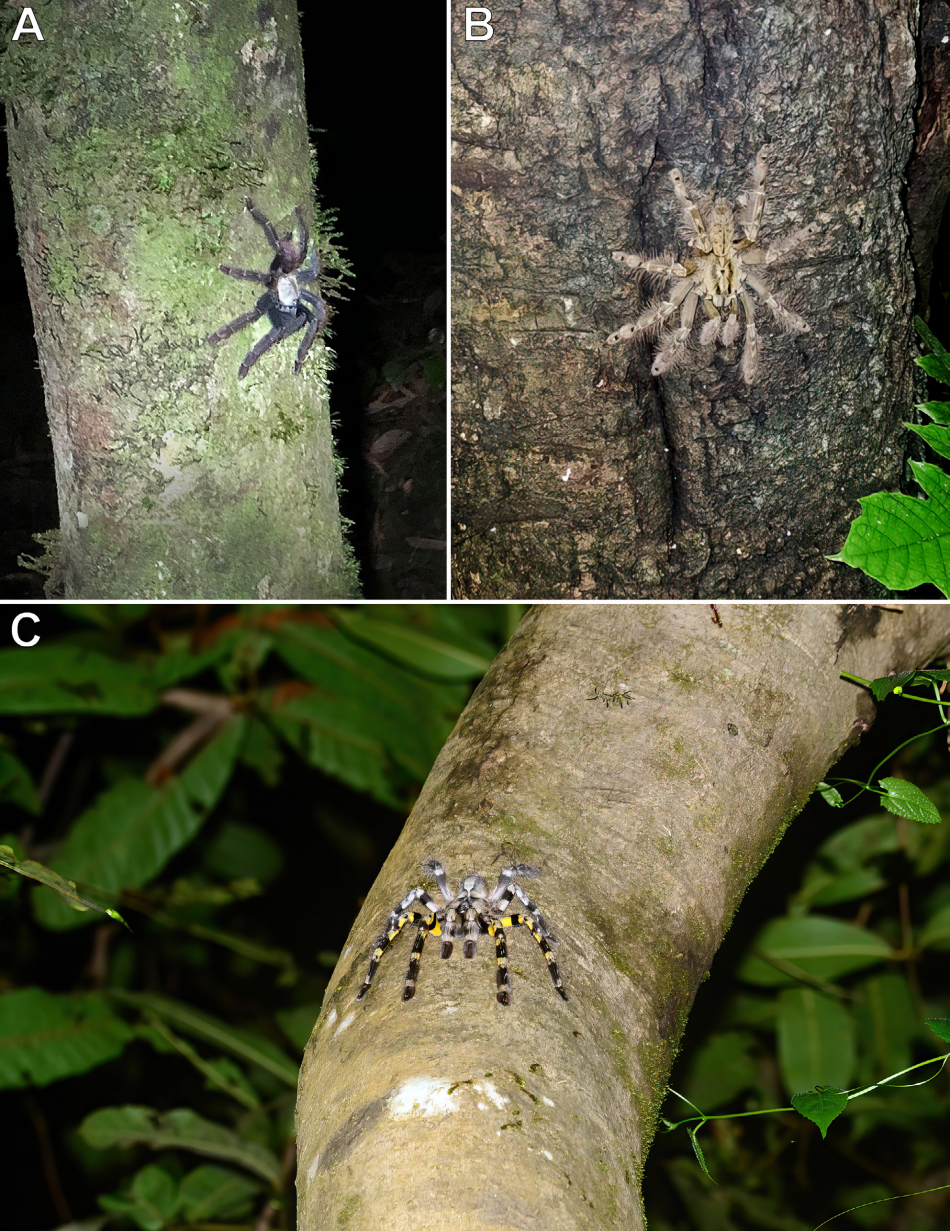
Arboreal tarantulas, adult or subadult females. (A) *Phormingochilus* sp.; Lahad Datu, Sabah, Borneo. (B) *Stromatopelma calceatum* (Fabricius, 1793); Man, Ivory Coast. (C) *Poecilotheria regalis* Pocock, 1899; Murud, Raigad, Roha, Maharashtra, India. Photos: Raphael S. von Büren (A), Johanna Fabiani (B), and Sushant More (C; reproduced under CC0 1.0, iNaturalist observation 59151113).

Nine cases of spatial orientation in arboreal and fossorial tarantulas in their natural environment were documented here. In general, arboreal tarantulas occupy a more limited vertical and circumferential space for navigation and hunting, in contrast to fossorial species that move across a largely horizontal, 360° plane. When active on trees or vertical man‐made structures at night, most arboreal tarantulas adopt a downward‐facing orientation (Figures 2A, 3B, 13A–C). This posture likely enhances spatial awareness and proprioceptive control, allowing them to more effectively capture prey that climbs from the forest floor toward the canopy. It may also facilitate faster strike movements by reducing gravitational resistance. Moreover, their more dorso‐ventrally flattened body form and stance, compared to the bulkier build of fossorial species, probably improve stability on rounded branches, trunks, and vertical surfaces, a capability further supported by densely scopulated feet that increase traction.

In the cases involving Aviculariinae, individuals were observed, on two occasions repeatedly, leaving their retreats to hunt at more favourable locations, such as areas near artificial light sources that attracted flying insects, located approximately 1–2 m away (Figure 2C). These observations suggest that the tarantulas may have learned, retained, and applied information from previous experiences to modify their foraging behaviour in ways that enhanced prey capture. This behaviour, known as spatial learning, has been reported in other arboreal tarantulas during nocturnal activity, with individuals travelling varying distances from retreats built in natural tree cavities, palm axils, tree trunks or anthropogenic structures to locations where prey availability was presumably higher (Charpentier 1992; Stradling 1994), as well as in several other groups of spiders (Punzo 2004).

**FIGURE 14:**
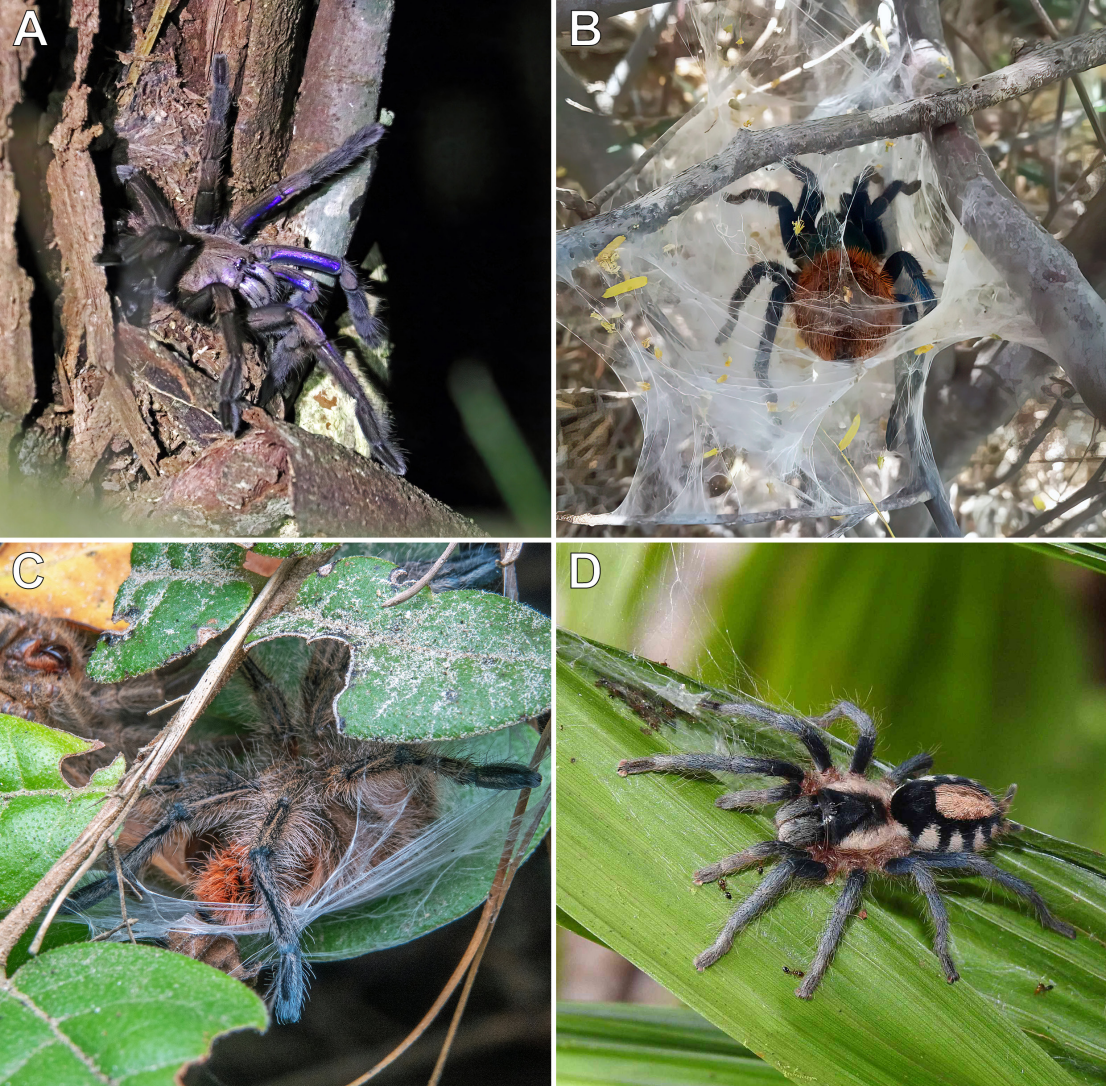
Fossorial tarantulas in arboreal microhabitats. (A) *Chilobrachys natanicharum* Chomphuphuang et al., 2023, adult female; Khao Sok National Park, Surat Thani, Thailand. (B) *Chromatopelma cyaneopubescens* (Strand, 1907), adult female; Paraguaná Peninsula, Falcón, Venezuela. (C) *Phrixotrichus* cf. *vulpinus* (Karsch, 1880), sex and life stage undetermined; Concepción, Bíobío, Chile. (D) *Cyriocosmus* sp., adult or subadult female; Trésor Regional Nature Reserve, Roura, French Guiana. Photos: Niran Anurakpongsathorn (A), Carlos Aramburu (B), Ernesto Guzman (C), and Olivier Fortune (D).

Somewhat analogous arboreal movements observed in otherwise fossorial species (observations #7 and #8), apparently associated with foraging in the tree canopy rather than on the ground during the dry season, further supports this interpretation. We also encountered several photographs on online platforms documenting similar arboreal movements and retreat construction in otherwise fossorial species from different genera and subfamilies (Figure 14A–D). These behaviours may represent similar ecological strategies, although further investigation is required. Temporary relocation to arboreal microhabitats may also be influenced by environmental conditions. In lowland floodplain areas, ground‐dwelling theraphosids, regardless of life stage, may climb and make temporary retreats in shrubs or trees during the rainy season to avoid seasonal inundation (West, pers. obs.). Similar responses to flooding have been documented in other invertebrates as well (e.g., Yamazaki et al. 2015).

**FIGURE 15:**
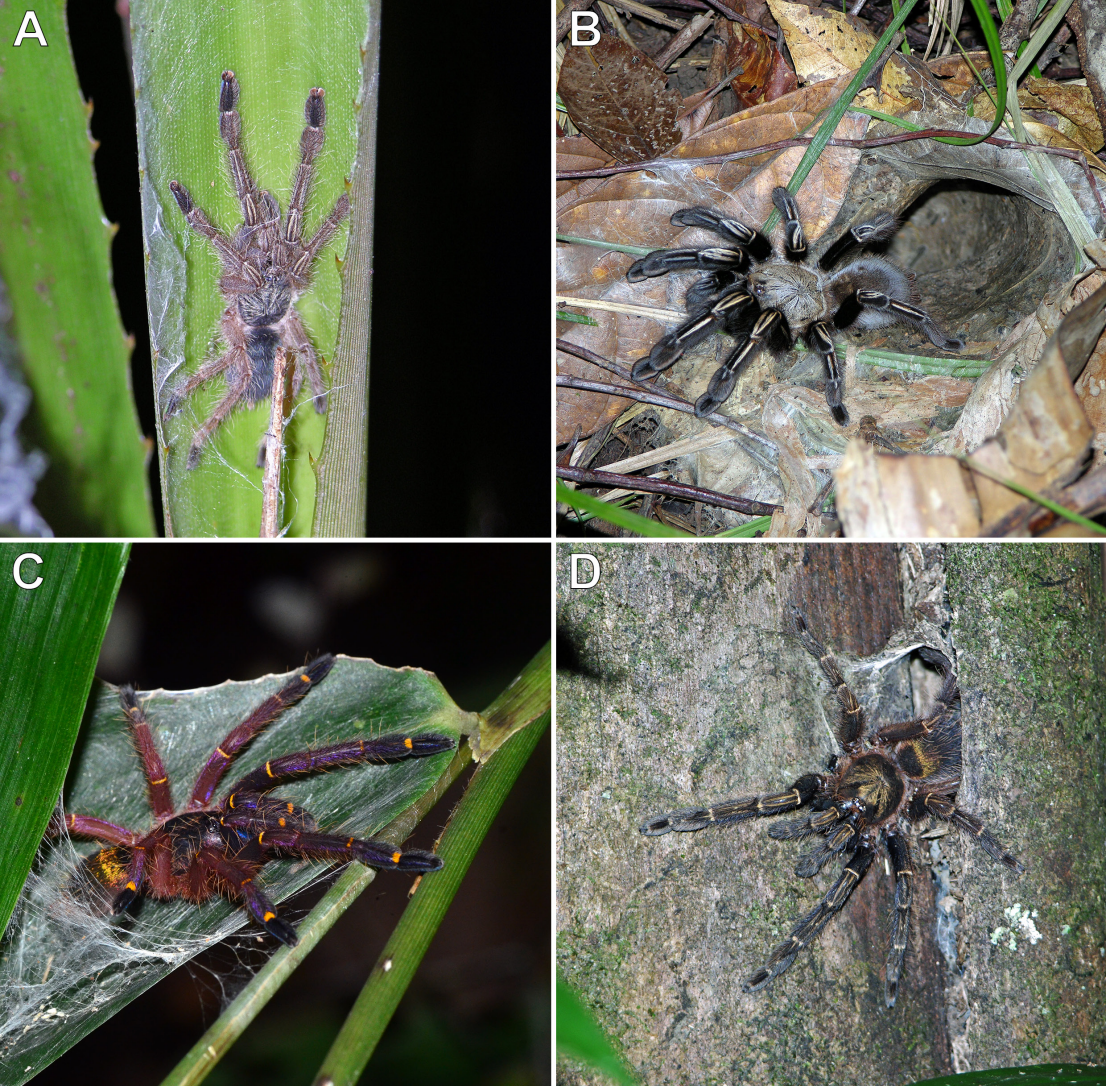
*Ephebopus* spp., juveniles and adult females. (A) *E. murinus* , juvenile, in an arboreal retreat; Montsinéry‐Tonnegrande, French Guiana. (B) *E. murinus* , adult female, outside a fossorial retreat; Montsinéry‐Tonnegrande, French Guiana. (C) *E. cyanognathus* , juvenile, in an arboreal retreat; Matoury, French Guiana. (D) *E. rufescens* , adult female, in an arboreal retreat; Montsinéry‐Tonnegrande, French Guiana. Photos: Rick C. West (A, B, D) and Ombeline Sculfort (C).

It should be noted that the apparently opportunistic behavioural plasticity observed in observations #7 and #8 differs from ontogenetic shifts in habitat use, in which different age‐ or size classes selectively utilise different microhabitats, possibly to reduce size‐dependent predation, cannibalism, and intraspecific competition (Werner and Gilliam 1984). Such shifts have been documented in several groups of spiders (e.g., Edgar 1971; Kronk and Riechert 1979; Rayor and Uetz 1993; Hazzi et al. 2025), including the theraphosid genera *Hysterocrates* Simon, 1892 and *Ephebopus* Simon, 1892 (West 2003; Marshall and West 2008). Of the former, which is a member of the entirely fossorial subfamily Eumenophorinae, juveniles of *H. crassipes* Pocock, 1897 found in large numbers in palms in Cameroon have been briefly described by West (2003). *Ephebopus*, in contrast, is a fossorial lineage within the otherwise arboreal subfamily Psalmopoeinae. Individuals of *E. cyanognathus* West & Marshall, 2000 and *E. murinus* (Walckenaer, 1837) occupy arboreal retreats during early life stages (Figure 15A,C) and later permanently move to the ground to construct fossorial retreats (Figure 15B) as prey demands increase (Marshall and West 2008; West, pers. obs.). Some adults of *E. rufescens* West & Marshall, 2000, however, were observed to remain in tree retreats, typically within root mass clusters in tree crotches (Figure 15D). Finally, on one occasion, a juvenile of the entirely fossorial theraphosine genus *Pamphobeteus* Pocock, 1901 was found in a self‐made silken retreat above ground among foliage in Peru (West, pers. obs.).

The remaining observations involve tarantulas quickly and directly returning to their retreats when threatened, without hesitation or disorientation. Studies on the catch‐and‐return behaviour of the funnel‐weaver *Agelena labyrinthica* (Clerck, 1757) (Agelenidae) under controlled conditions (e.g., Bartels 1929; Görner 1962; Görner and Claas 1985) suggest that navigation in this species primarily relies on a combination of external and internal cues acquired during the outbound journey, including light position, polarised light, web tension patterns, gravity, and idiothetic signals; alternatively, the spider may rely only on idiothetic information, even when external cues are available (Moller 1970; Görner 1972). The close integration of allothetic and idiothetic cues suggests that both types of information are processed within the same region of the central nervous system (Foelix 2025).

A more comparable case may be the nocturnal wandering spider *Cupiennius salei* (Keyserling, 1877) (Trechaleidae). Blinded individuals can accurately return to a prey capture site after displacement by relying on idiothetic cues, particularly input from slit sensilla (lyriform organs when compound), which are mechanoreceptors near the leg joints that detect exoskeletal strain (Barth 2004). Spiders with mechanically damaged lyriform organs showed reduced return accuracy, greater navigational errors, and an inability to navigate curved paths (Barth and Seyfarth 1971; Seyfarth and Barth 1972; Seyfarth et al. 1982). We report a similar case in juveniles of *H. sprousei* , a blind troglobitic tarantula, which retained awareness of both the direction and distance to their retreats in the absence of vision or polarised light. It is noteworthy that adults of *H. sprousei* and other troglobitic congeners in different cave systems display more erratic movement patterns, with no fixed retreats except when mated females construct hammock‐like egg sacs (Figure 12B) within natural rock cavities (Mendoza and Francke 2018). This suggests a possible ontogenetic shift in foraging behaviour, with reduced reliance on fixed retreats as individuals mature and the energetic demands of capturing larger prey increase.

It remains unclear whether tarantulas use both allothetic and idiothetic cues to navigate back to their retreats. In *A. labyrinthica* and *C. salei* , navigation appears to rely mainly on idiothetic cues, as noted above. In contrast, in the wolf spider *Lycosa tarantula* (Linnaeus, 1758) (Lycosidae), blinding the anterior median eyes, which detect polarised light, disrupts return to the retreat, which indicates a high reliance on this allothetic cue for navigation (Ortega‐Escobar and Muñoz‐Cuevas 1999). Given that most tarantulas are predominantly nocturnal, idiothetic cues are likely more important, though they are capable of detecting polarised light (Henton and Crawford 1966), which may be useful during the less frequent diurnal returns. Silk draglines left along the path back to the burrow also aid navigation, as discussed further below (Minch 1978; Shillington and Verrell 1997; Gaffin and Curry 2020).

While efficient in short‐range navigation, tarantulas seemingly lack the ability for long‐distance path integration seen in a few other groups. For example, adult males of the wandering huntsman *Leucorchestris arenicola* Lawrence, 1962 (Sparassidae) undertake nocturnal round trips of several tens of meters to locate females and return directly to their burrows, likely by using local cues (Nørgaard et al. 2007). Mate‐searching excursions in tarantulas, on the other hand, are almost entirely random and do not circle back to a central location (Janowski‐Bell and Horner 1999).

Instincts are innate, inherited behaviour patterns essential for survival, guiding actions such as migration, hunting, mating, and nest‐building without prior learning. They are triggered by specific environmental cues to produce consistent, adaptive responses. Learning, in contrast, is the process by which experience modifies behaviour, often permanently, enabling animals to adapt to changing conditions. Spiders, long considered largely instinct‐driven animals, in fact exhibit remarkable learning, memory, and behavioural plasticity despite their tiny brains. Associative learning in spiders has been documented in different contexts, such as linking vibration frequency (Bays 1962) or colouration (Jakob et al. 2007) to prey taste, navigating modified webs and improving return paths after repeated trials (LeGuelte 1969), remembering previously captured (Rodriguez and Gamboa 2000) or temporarily out‐of‐sight prey while adjusting their movements based on past experiences and accounting for changes in their own position (Hill 1979; Tarsitano and Jackson 1992, 1994), and searching for separated egg sacs, which they will accept within 1–2 days (Peckham and Peckham 1887), all of which reflect the presence of short‐ and long‐term memory and cognitive processes. Learning can be strong enough to even override innate preferences, as shown in specialised myrmecophagous spiders that, when raised on detrimental prey, such as fruit flies, come to prefer these over their naturally optimal ant prey (Pekař and Cárdenas 2015).

Most experiments on the learning capacities of spiders have focused on visual hunters (e.g., Lycosidae, Salticidae) and orb‐weavers (e.g., Araneidae), while other groups, such as tarantulas, have received little attention; this is likely due to their generally sedentary nature, despite the potential for species with longer lifespans, such as tarantulas, to evolve more flexible behavioural programs (Poli 1988). The few studies conducted on tarantulas have demonstrated both spatial reversal and complex maze learning, which require a neural architecture more complex than that found in most arthropods (Gallistel 1990). In a T‐maze, most individuals learned over repeated trials to avoid bright light and heat (Punzo 2002) or to orient toward polarised light (Henton and Crawford 1966). In a complex six‐alley maze, most learned to navigate efficiently, making progressively fewer errors and reaching the goal more quickly after 10 days of training (Punzo 2002). In nature, individuals inhabiting abandoned rodent burrows, which often have multiple side branches, adjust their position and egg sac placement according to temperature and humidity (Main 1982; Kotzman 1990), which indicate their ability to remember different sites in three‐dimensional space for survival and reproduction (Punzo 2002). According to another experiment, *A. chalcodes* can learn and retain avoidance behaviours, such as adjusting leg position to avoid an aversive stimulus, which is associated with increased RNA and protein synthesis in the supraesophageal ganglion (SEG) and decreased acetylcholinesterase activity. Blocking protein synthesis with cycloheximide impairs learning, particularly when applied before training. RNA activity rises specifically in the protocerebrum and central body of the SEG, but not in the optic or subesophageal ganglia, which demonstrates the role of the SEG in learning (Punzo 1988).

Other research has revealed additional cognitive complexities in tarantulas, including behavioural laterality (Ruhland et al. 2017), as well as physiological and behavioural changes indicative of stress or altered internal states, which may interact with cognitive processes. These include increased levels of hemolymphal cortisol, a hormone associated with chronic stress, and a reduced range of behavioural patterns under full‐spectrum lighting (Somerville et al. 2021), decreased levels of serotonin and octopamine in the SEG following agonistic interactions between males, particularly in subordinates (Punzo and Punzo 2001), highly variable durations of tonic immobility in ‘flipped’ tarantulas, where massed trials produced more prolonged responses consistent with elevated fear or stress‐like states (Crawford 1979), and increases in aggression and flight responses in individuals raised in unenriched enclosures, whereas enriched conditions result in more ‘positive’ behaviours (Bennie et al. 2011).

Although the behavioural cases described suggest that learning and memory may contribute to prey‐searching and navigation, the cognitive interpretation proposed here should be regarded as preliminary in the absence of experimental evidence. As noted, mygalomorphs rely extensively on chemical and chemo‐tactile cues, particularly those associated with silk, which may provide alternative or complementary explanations for retreat recognition and foraging movements (Yáñez et al. 1999; Dor et al. 2008; Copperi et al. 2019). For example, movement of juvenile tarantulas around the maternal burrow indicates that peripheral silk networks provide a cue‐bearing substrate that helps maintain spatial position and facilitates returning to the retreat (Shillington and McEwen 2006). Silk‐borne signals are also important in reproductive behaviours and sexual communication, allowing males to locate females, assess their reproductive status, and initiate courtship near burrows (Minch 1979; Quirici and Costa 2005; Copperi et al. 2019).

Prey‐searching behaviour in spiders likewise depends on multiple sensory modalities, including chemical, vibrational, and visual cues (Uetz et al. 2013). Chemical traces left by prey can influence microhabitat selection and patch residence time, while visual features such as habitat structure and light availability may further affect site choice (Persons and Uetz 1996a, 1996b; Punzo and Kukoyi 1997; Persons and Rypstra 2000; de Omena and Romero 2010). For example, in human‐modified environments, artificial lighting often creates stable prey‐rich patches that spiders preferentially forage in (Heiling 1999; Mammola et al. 2018), which may partly explain the behaviours reported in observations #1 and #3.

Overall, available evidence suggests that learning‐based navigation and cue‐based orientation are not mutually exclusive and may interact in shaping movement and foraging behaviour in spiders. While the observations reported here are compatible with experience‐based spatial behaviour, they may also be explained, at least in part, by well‐documented sensory mechanisms. Combining field observations with manipulative experiments designed to test learning hypotheses under controlled conditions will therefore be essential for assessing the relative contributions of cognitive and sensory processes to orientation and foraging in mygalomorph spiders.

## Author Contributions

**Alireza Zamani:** conceptualization (equal), investigation (equal), resources (supporting), visualization (equal), writing – original draft (lead), writing – review and editing (lead). **Rick C. West:** conceptualization (equal), investigation (equal), resources (lead), visualization (equal), writing – original draft (supporting), writing – review and editing (supporting).

## Conflicts of Interest

The authors declare no conflicts of interest.

## Data Availability

No datasets were generated or analysed during the current study.

## References

[ece373329-bib-0001] Bartels, M. 1929. “Sinnesphysiologische und psychologische Untersuchungen an der Trichterspinne *Agelena labyrinthica* (Cl.).” Zeitschrift für Vergleichende Physiologie 10: 527–593.

[ece373329-bib-0002] Barth, F. G. 2002. A Spider's World: Senses and Behavior, 394. Springer‐Verlag.

[ece373329-bib-0003] Barth, F. G. 2004. “Spider Mechanoreceptors.” Current Opinion in Neurobiology 14, no. 4: 415–422. 10.1016/j.conb.2004.07.005. 15321061

[ece373329-bib-0004] Barth, F. G. , and E.‐A. Seyfarth . 1971. “Slit Sense Organs and Kinesthetic Orientation.” Zeitschrift für Vergleichende Physiologie 74: 326–328.

[ece373329-bib-0005] Bays, S. M. 1962. “A Study on the Training Possibilities of *Araneus diadematus* Cl.” Experientia (Basel) 18: 423. 13967102 10.1007/BF02151499

[ece373329-bib-0006] Bennie, M. , C. Loaring , and S. Trim . 2011. “Laboratory Husbandry of Arboreal Tarantulas (Theraphosidae) and Evaluation of Environmental Enrichment.” Animal Technology 10: 163–169.

[ece373329-bib-0007] Charpentier, P. 1992. “The Genus *Avicularia* .” Exothermae 1, no. 1: 1–41.

[ece373329-bib-0008] Collett, T. S. 2019. “Path Integration: How Details of the Honeybee Waggle Dance and the Foraging Strategies of Desert Ants Might Help in Understanding Its Mechanisms.” Journal of Experimental Biology 222: jeb205187. 10.1242/jeb.205187. 31152122

[ece373329-bib-0009] Coons, D. 1976. “Cueva de la Laguna Verde.” Canadian Caver 8, no. 2: 18–23.

[ece373329-bib-0010] Copperi, M. S. , N. Ferretti , and A. V. Peretti . 2019. “The Role of Silk in Courtship and Communication in Mygalomorph Spiders: Do Males Regulate Their Courtship in Response to Female Mating Status?” Behavioural Processes 167: 103939. 10.1016/j.beproc.2019.103939. 31421152

[ece373329-bib-0011] Crawford, F. T. 1979. “The Effect of Distribution of Trials Upon the Habituation of Tonic Immobility in the Tarantula, *Aphonopelma californica* .” Bulletin of the Psychonomic Society 14: 135–137.

[ece373329-bib-0012] de Omena, P. M. , and G. Q. Romero . 2010. “Using Visual Cues of Microhabitat Traits to Find Home: The Case Study of a Bromeliad‐Living Jumping Spider (Salticidae).” Behavioral Ecology 21: 690–695. 10.1093/beheco/arq040.

[ece373329-bib-0013] Dor, A. , S. Machkour‐M’Rabet , L. Legal , T. Williams , and Y. Hénaut . 2008. “Chemically Mediated Burrow Recognition in the Mexican Tarantula *Brachypelma vagans* Female.” Naturwissenschaften 95, no. 12: 1189–1193. 10.1007/s00114-008-0441-5. 18712335

[ece373329-bib-0014] Edgar, W. D. 1971. “The Life‐Cycle, Abundance and Seasonal Movement of the Wolf Spider, *Lycosa (Pardosa) lugubris*, in Central Scotland.” Journal of Animal Ecology 40: 303–322.

[ece373329-bib-0015] Elliott, W. R. 2020. “The Catfish Caves of Acatlán, Oaxaca Reviving Cave Surveys From Long Ago.” AMCS Activities Newsletter 42: 22–30.

[ece373329-bib-0016] Foelix, R. 2025. Spider Biology. 1st ed, 470. Springer.

[ece373329-bib-0017] Gaffin, D. D. , and C. M. Curry . 2020. “Arachnid Navigation—A Review of Classic and Emerging Models.” Journal of Arachnology 48, no. 1: 1–25. 10.1636/0161-8202-48.1.1.

[ece373329-bib-0018] Gallistel, C. R. 1990. The Organization of Learning. MIT Press.

[ece373329-bib-0019] Görner, P. 1962. “Die Orientierung der Trichterspinne nach polarisiertem Licht.” Zeitschrift für Vergleichende Physiologie 45: 307–314.

[ece373329-bib-0020] Görner, P. 1972. “Resultant Positioning Between Optical and Kinesthetic Orientation in the Spider *Agelena labyrinthica* Clerck.” In Information Processing in the Visual Systems of Arthropods, edited by R. Wehner , 269–274. Springer.

[ece373329-bib-0021] Görner, P. , and B. Claas . 1985. “Homing Behavior and Orientation in the Funnel‐Web Spider, *Agelena labyrinthica* Clerck.” In Neurobiology of Arachnids, edited by F. G. Barth , 275–297. Springer.

[ece373329-bib-0022] Hazzi, N. A. , H. M. Wood , and G. Hormiga . 2025. “Repeated Habitat Shifts and Varying Dispersal Rates Between Habitats Shape Ecomorphological Assembly of Wandering Ctenidae Spiders Across Continents.” Journal of Evolutionary Biology 38, no. 9: 1218–1232. 10.1093/jeb/voaf074. 40489325

[ece373329-bib-0023] Heiling, A. 1999. “Why Do Nocturnal Orb‐Web Spiders (Araneidae) Search for Light?” Behavioral Ecology and Sociobiology 46: 43–49. 10.1007/s002650050590.

[ece373329-bib-0024] Henton, W. W. , and F. T. Crawford . 1966. “The Discrimination of Polarized Light by the Tarantula.” Zeitschrift für Vergleichende Physiologie 52: 26–32.

[ece373329-bib-0025] Hill, D. E. 1979. “Orientation by Jumping Spiders of the Genus *Phidippus* (Araneae: Salticidae) During Pursuit of Prey.” Behavioral Ecology and Sociobiology 5: 301–322.

[ece373329-bib-0026] Jakob, E. M. , C. D. Skow , M. P. Haberman , and A. Plourde . 2007. “Jumping Spiders Associate Food With Color Cues in a T‐Maze.” Journal of Arachnology 35, no. 3: 487–492. 10.1636/JOA-ST06-61.1.

[ece373329-bib-0027] Janowski‐Bell, M. E. , and N. V. Horner . 1999. “Movement of the Male Brown Tarantula, *Aphonopelma hentzi* (Araneae, Theraphosidae), Using Radio Telemetry.” Journal of Arachnology 27, no. 3: 503–512.

[ece373329-bib-0028] Japyassu, H. F. , and K. N. Laland . 2017. “Extended Spider Cognition.” Animal Cognition 20: 375–395. 10.1007/s10071-017-1069-7. 28176133 PMC5394149

[ece373329-bib-0029] Kotzman, M. 1990. “Annual Activity Patterns of the Australian Tarantula *Selenocosmia stirlingi* (Araneae, Theraphosidae) in an Arid Area.” Journal of Arachnology 18, no. 1: 123–130.

[ece373329-bib-0030] Kronk, A. E. , and S. E. Riechert . 1979. “Parameters Affecting the Habitat Choice of a Desert Wolf Spider, *Lycosa santrita* Chamberlin and Ivie.” Journal of Arachnology 7, no. 2: 155–166.

[ece373329-bib-0031] LeGuelte, L. 1969. “Learning in Spiders.” American Zoologist 9: 145–152. 5363237 10.1093/icb/9.1.145

[ece373329-bib-0032] Main, B. Y. 1982. “Adaptations to Arid Habitats by Mygalomorph Spiders.” In Evolution of the Flora and Fauna of Arid Australia, edited by W. R. Barker and P. J. M. Greenslade , 273–283. Peacock Publishing.

[ece373329-bib-0033] Mammola, S. , M. Isaia , D. Demonte , P. Triolo , and M. Nervo . 2018. “Artificial Lighting Triggers the Presence of Urban Spiders and Their Webs on Historical Buildings.” Landscape and Urban Planning 180: 187–194. 10.1016/j.landurbplan.2018.09.003.

[ece373329-bib-0034] Marshall, S. D. , and R. West . 2008. “An Ontogenetic Shift in Habitat Use by the Neotropical Tarantula *Ephebopus murinus* (Araneae, Theraphosidae, Aviculariinae).” Arachnology 14, no. 6: 280–284. 10.13156/arac.2011.14.6.280.

[ece373329-bib-0035] Mendoza, J. I. , and O. F. Francke . 2018. “Five New Cave‐Dwelling Species of *Hemirrhagus* Simon 1903 (Araneae, Theraphosidae, Theraphosinae), With Notes on the Generic Distribution and Novel Morphological Features.” Zootaxa 4407, no. 4: 451–482. 10.11646/zootaxa.4407.4.1. 29690167

[ece373329-bib-0036] Minch, E. W. 1978. “Daily Activity Patterns in the Tarantula *Aphonopelma chalcodes* Chamberlin.” Bulletin of the British Arachnological Society 4: 231–237.

[ece373329-bib-0037] Minch, E. W. 1979. “Reproductive Behaviour of the Tarantula *Aphonopelma chalcodes* Chamberlin (Araneae: Theraphosidae).” Bulletin of the British Arachnological Society 4, no. 9: 416–420.

[ece373329-bib-0038] Moller, P. 1970. “Die Systematischen Abweichungen bei der optischen Richtungsorientierung der Trichterspinne *Agelena labyrinthica* .” Zeitschrift für Vergleichende Physiologie 66: 78–106.

[ece373329-bib-0039] Nørgaard, T. 2005. “Nocturnal Navigation in *Leucorchestris arenicola* (Araneae: Sparassidae).” Journal of Arachnology 33, no. 2: 533–540. 10.1636/04-113.1.

[ece373329-bib-0040] Nørgaard, T. , J. R. Henschel , and R. Wehner . 2007. “Use of Local Cues in the Night‐Time Navigation of the Wandering Desert Spider *Leucorchestris arenicola* (Araneae, Sparassidae).” Journal of Comparative Physiology A 193: 217–222. 10.1007/s00359-006-0178-6. 17235606

[ece373329-bib-0041] Ortega‐Escobar, J. 2020. “Homing in the Arachnid Taxa Araneae and Amblypygi.” Animal Cognition 23, no. 3: 1189–1204. 10.1007/s10071-020-01424-w. 32894371

[ece373329-bib-0042] Ortega‐Escobar, J. , and A. Muñoz‐Cuevas . 1999. “Anterior Median Eyes of *Lycosa tarentula* [Sic!] (Araneae, Lycosidae) Detect Polarized Light: Behavioral Experiments and Electroretinographic Analysis.” Journal of Arachnology 27, no. 3: 663–671.

[ece373329-bib-0043] Peckham, G. W. , and E. G. Peckham . 1887. “Some Observations on the Mental Powers of Spiders.” Journal of Morphology and Physiology 1: 383–419.

[ece373329-bib-0044] Pekař, S. , and M. Cárdenas . 2015. “Innate Prey Preference Overridden by Familiarisation With Detrimental Prey in a Specialised Myrmecophagous Predator.” Science of Nature 102: 8. 10.1007/s00114-015-1257-8. 25645732

[ece373329-bib-0045] Persons, M. H. , and A. L. Rypstra . 2000. “Preference for Chemical Cues Associated With Recent Prey in the Wolf Spider *Hogna helluo* (Araneae: Lycosidae).” Ethology 106, no. 1: 27–35. 10.1046/j.1439-0310.2000.00496.x.

[ece373329-bib-0046] Persons, M. H. , and G. W. Uetz . 1996a. “The Influence of Sensory Information on Patch Residence Time in Wolf Spiders (Araneae: Lycosidae).” Animal Behaviour 51, no. 6: 1285–1293. 10.1006/anbe.1996.0133.

[ece373329-bib-0047] Persons, M. H. , and G. W. Uetz . 1996b. “Wolf Spiders Vary Patch Residence Time in the Presence of Chemical Cues From Prey (Araneae, Lycosidae).” Journal of Arachnology 24, no. 1: 76–79.

[ece373329-bib-0048] Poli, M. D. 1988. “Species‐Specific Differences in Learning.” In Intelligence and Evolutionary Biology, edited by H. J. Jerison and L. Jerison , 277–297. Springer‐Verlag.

[ece373329-bib-0049] Punzo, F. 1988. “Learning and Localization in Brain Function in the Tarantula Spider, *Aphonopelma chalcodes* (Orthognatha, Theraphosidae).” Comparative Biochemistry and Physiology Part A: Physiology 89: 465–470.

[ece373329-bib-0050] Punzo, F. 2002. “Reversal Learning and Complex Maze Learning in the Spider *Aphonopelma hentzi* (Girard) (Araneae, Theraphosidae).” Bulletin of the British Arachnological Society 12: 153–158.

[ece373329-bib-0051] Punzo, F. 2004. “The Capacity for Spatial Learning in Spiders: A Review.” Bulletin of the British Arachnological Society 13, no. 3: 65–72.

[ece373329-bib-0052] Punzo, F. , and O. Kukoyi . 1997. “The Effects of Prey Chemical Cues on Patch Residence Time in the Wolf Spider *Trochosa parthenus* (Chamberlin) (Lycosidae) and the Lynx Spider *Oxyopes salticus* Hentz (Oxyopidae).” Bulletin of the British Arachnological Society 10, no. 9: 323–326.

[ece373329-bib-0053] Punzo, F. , and T. Punzo . 2001. “Monoamines in the Brain of Tarantulas ( *Aphonopelma hentzi* ) (Araneae, Theraphosidae): Differences Associated With Male Agonistic Interactions.” Journal of Arachnology 29, no. 3: 388–395.

[ece373329-bib-0054] Quirici, V. , and F. G. Costa . 2005. “Seismic Communication During Courtship in Two Burrowing Tarantula Spiders: An Experimental Study on *Eupalaestrus weijenberghi* and *Acanthoscurria suina* .” Journal of Arachnology 33, no. 1: 159–166. 10.1636/S03-22.

[ece373329-bib-0055] Rayor, L. S. , and G. W. Uetz . 1993. “Ontogenetic Shifts Within the Selfish Herd: Predation Risk and Foraging Trade‐Offs Change With Age in Colonial Web‐Building Spiders.” Oecologia 95, no. 1: 1–8. 28313304 10.1007/BF00649499

[ece373329-bib-0056] Reddell, J. 1981. “A Review of the Cavernicole Fauna of Mexico, Guatemala and Belize.” Bulletin of the Texas Memorial Museum (Association for Mexican Cave Studies) 27: 1–327.

[ece373329-bib-0057] Rodriguez, R. L. , and E. Gamboa . 2000. “Memory of Captured Prey in Three Web Spiders (Araneae: Araneidae, Linyphiidae, Tetragnathidae).” Animal Cognition 3: 91–97. 10.1007/s100710000066.

[ece373329-bib-0058] Ruhland, F. , J.‐P. Caudal , C. Blois‐Heulin , and M. Trabalon . 2017. “Male Tarantula Spiders' Reactions to Light and Odours Reveal Their Motor Asymmetry.” Journal of Zoology 301: 51–60. 10.1111/jzo.12388.

[ece373329-bib-0059] Seyfarth, E.‐A. , and F. G. Barth . 1972. “Compound Slit Sense Organs on the Spider Leg: Mechanoreceptors Involved in Kinesthetic Orientation.” Journal of Comparative Physiology 78: 176–191.

[ece373329-bib-0060] Seyfarth, E.‐A. , R. Hergenröder , H. Ebbes , and F. G. Barth . 1982. “Idiothetic Orientation of a Wandering Spider: Compensation of Detours and Estimates of Goal Distance.” Behavioral Ecology and Sociobiology 11: 139–148.

[ece373329-bib-0061] Shillington, C. , and B. McEwen . 2006. “Activity of Juvenile Tarantulas in and Around the Maternal Burrow.” Journal of Arachnology 34, no. 1: 261–265.

[ece373329-bib-0062] Shillington, C. , and P. Verrell . 1997. “Sexual Strategies of a North American ‘Tarantula’ (Araneae: Theraphosidae).” Ethology 103: 588–598.

[ece373329-bib-0063] Shorthouse, D. 2010. SimpleMappr, an Online Tool to Produce Publication‐Quality Point Maps. http://www.simplemappr.net.

[ece373329-bib-0064] Somerville, S. , S. Baker , F. Baines , S. A. Trim , and C. Trim . 2021. “Full Spectrum Lighting Induces Behavioral Changes and Increases Cortisol Immunoreactivity in Captive Arachnids.” Journal of Applied Animal Welfare Science 24, no. 2: 132–148. 10.1080/10888705.2021.1872027. 33559500

[ece373329-bib-0065] Stradling, D. J. 1994. “Distribution and Behavioural Ecology of an Arboreal Tarantula Spider in Trinidad.” Biotropica 26: 84–97.

[ece373329-bib-0066] Tarsitano, M. S. , and R. R. Jackson . 1992. “Influence of Prey Movement on the Performance of Simple Detours by Jumping Spiders.” Behaviour 123, no. 1–2: 106–120. 10.1163/156853992X00147.

[ece373329-bib-0067] Tarsitano, M. S. , and R. R. Jackson . 1994. “Jumping Spiders Make Predatory Detours Requiring Movement Away From Prey.” Behaviour 131, no. 1–2: 65–73. 10.1163/156853994X00217.

[ece373329-bib-0068] Uetz, G. W. , J. A. Roberts , D. L. Clark , J. S. Gibson , and S. D. Gordon . 2013. “Multimodal Signals Increase Active Space of Communication by Wolf Spiders in a Complex Litter Environment.” Behavioral Ecology and Sociobiology 67: 1471–1482. 10.1007/s00265-013-1557-y.

[ece373329-bib-0069] Werner, E. E. , and J. F. Gilliam . 1984. “The Ontogenetic Niche and Species Interactions in Size‐Structured Populations.” Annual Review of Ecology and Systematics 15: 393–425.

[ece373329-bib-0070] West, R. C. 2003. “Cameroon Adventure and ‘Spider Divination’.” Journal of the British Tarantula Society 18, no. 3: 76–84.

[ece373329-bib-0071] Yamazaki, L. , M. I. Marques , A. D. Brescovit , and L. D. Battirola . 2015. “ *Tityus paraguayensis* (Scorpiones: Buthidae) em Copas de *Callisthene fasciculata* (Vochysiaceae) no Pantanal Mato Grosso (Brasil).” Acta Biológica Paranaense 44: 1–4. 10.5380/abpr.v44i1-4.44122.

[ece373329-bib-0072] Yáñez, M. , A. Locht , and R. Macías‐Ordóñez . 1999. “Courtship and Mating Behavior of *Brachypelma klaasi* (Araneae, Theraphosidae).” Journal of Arachnology 27, no. 1: 165–170.

